# Predicting miRNA-disease associations via layer attention graph convolutional network model

**DOI:** 10.1186/s12911-022-01807-8

**Published:** 2022-03-19

**Authors:** Han Han, Rong Zhu, Jin-Xing Liu, Ling-Yun Dai

**Affiliations:** School of Computer Science, Qufu Normal University, Rizhao, China

**Keywords:** MiRNA-disease associations, Layer attention, Graph convolution network, Predict

## Abstract

**Background:**

MiRNA is a class of non-coding single-stranded RNA molecules with a length of approximately 22 nucleotides encoded by endogenous genes, which can regulate the expression of other genes. Therefore, it is very important to predict the associations between miRNA and disease. Predecessors developed a new prediction method of drug-disease association, and it achieved good results.

**Methods:**

In this paper, we introduced the method of LAGCN to identify potential miRNA-disease associations. First, we integrate three associations into a heterogeneous network, such as the known miRNA-disease association, miRNA-miRNA similarities and disease-disease similarities, next we apply graph convolution network to learn the embedding of miRNA and disease. We use an attention mechanism to combine embedding from multiple convolution layers. Unobserved miRNA-disease associations are scored based on integrated embedding.

**Results:**

After fivefold cross-validations, the value of AUC is reached 0.9091, which is higher than other prediction methods and baseline methods.

**Conclusions:**

In this paper, we introduced the method of LAGCN to identify potential miRNA-disease associations. LAGCN has achieved good performance in predicting miRNA-disease associations, and it is superior to other association prediction methods and baseline methods.

## Background

MicroRNAs (miRNAs) is small non-coding RNA, and it can influence the expression of their mRNA targets. MiRNA affects the expression of mRNA mainly by controlling RNA cleavage or translation inhibition [1]. A lot of experiments confirmed that miRNAs are important in many biological processes, such as cell development, proliferation, differentiation, death, metabolism, aging, apoptosis, signal transduction, and viral infection [2]. According to the relevant biological studies, it has been shown that there is a complicated association between the disorder of miRNAs and the occurrence and development of complex diseases. There has been a lot of evidence about that miRNAs are important for the diagnosis of heart disease and eventually cure it [3], and other serious diseases like malignancies, cardiovascular, mental disorders and diabetes. So far, it has been confirmed that many microRNAs are associated with cancer. Identification of miRNA-disease associations is of great significance for the diagnosis of related diseases and eventually cure them. Hence, researchers have conducted a lot of experiments in order to develop better methods to predict associations between miRNA and disease in the future [4].

In the past period, lots of studies have shown that there are some close associations between miRNAs and diseases [5]. Recently, many miRNAs have been found in various living organisms [6]. But as the study progressed, the researchers found that previous methods were time-consuming and expensive. Hence, researchers need more convenient and relatively cheaper research methods.

During the past few years, researchers have proposed a large number of methods for predicting miRNA and disease associations [7], and these methods are based on computational algorithms.

Liu et al. [8] developed a neighborhood-based computing model to predict the potential association between miRNA and disease. They used 50 miRNAs about breast cancer, esophageal cancer and colon cancer respectively, and which 48, 47 and 48 miRNAs were successfully confirmed. This shows that the method studied by Li et al. is feasible. They mainly used the K-nearest neighbor (KNN) method to improve the prediction accuracy of the associations between miRNA and disease.

Shi et al. [9] proposed a new calculation method. This new method uses the association between miRNAs and disease genes to randomly walk through the protein–protein interaction network to calculate a specific value. They assessed the size of this value tod etermine the association between miRNA and disease. They set a threshold, and if this value exceeds the threshold, the corresponding miRNA is considered to be associated with the disease, otherwise, they are considered miRNA is not to be associatedw ith the disease.

Zeng et al. [10] sorted out the existing calculation methods and divided them into two categories. The first method is the similarity measure-based prediction. A large number of the computational methods are based on the assumption that miRNAs with similar functions are more likely to be associated with phenotypically similar diseases and vice versa [2]. For example, predecessors developed a new method about the random walk to infer potential miRNA-disease interactions. And this method achieves this goal by realizing a random walk on a network about miRNA similar functions [2]. Li et al. [1] developed a new method about similarity network fusion and inductive matrix completion to predict miRNA-disease associations. And the second method is machine learning-based predictions.

Both of these methods work well, but there are still some drawbacks. A lot of end-to-end methods have been presented due to the development of deep learning techniques [11]. In order to get a better method. The novel prediction method is better than previous ones.

Graph convolutional networks have achieved good results in other fields. Chen et al. [11] presented a new method for structure-aware protein solubility prediction. The method predicts protein solubility by combining GCN with the predicted contact graph, and this method works well. Fang et al. presented a method to discover non-small cell lung cancer complexity across data modalities impacting IO benefit combined with GCN. It is an opportunity to use graph AI modeling for precision oncology [12]. Wang et al. [13] presented a new prediction method for graph convolutional networks. Simulation results illustrate that the proposed method has high performance.

Ana B. O. V. Silva and E. J. Spinosa proposed a graph convolution auto-encoder to predict lncRNA-disease associations. This method has been shown that AUC-ROC achieved 0.976, and this method is the most advanced method to solve the same problem [14]. Since graph convolutional network can achieve good results in lncRNA-disease associations prediction, it can also make some achievements in miRNA-disease association prediction.

Li et al. [15] proposed a method to identify the transmission source of infectious diseases using a graph convolutional neural network. Graph convolutional network is a method that combines graphs with deep learning.

Wang et al. [16] proposed a method about graph convolutional network model (CGINet) in order to identify chemical gene interactions in synthetic polygraphs. CGINet trains encoders and decoders mainly through end-to-end and through known chemical-gene interactions, and it gets a good grade.

Although GCN has achieved good results in other areas, it is rarely used in miRNA-disease associations prediction.

## Materials and methods

### GCN overview

In this section, we present the methodology of our graph convolutional networks. Convolution neural networks (CNN) had a property called translation invariance, which does not apply to the non-matrix structure, so the convolutional network of this graph appears timely. Graph convolutional networks were first introduced to processing graph structure data. First, we can describe the graph as:

1 $$G = (V,E),$$

where $V$ represents the node-set of the graph and $E$ represents the edge set of the graph. From the previously hidden layer to the next hidden layer, feature transformation is carried out on the nodes.

2 $$X^{(l + 1)} = f\left( {X^{(l)} ,A} \right),$$

where $X^{(l)}$ is the feature of layer l, and $A$ is the adjacency matrix. Expand Formula (2), and get Formula (3):

3 $$X^{(l + 1)} = \sigma \left( {AX^{(l)} W^{(l)} + b^{(l)} } \right),$$

where $\sigma$ represents a nonlinear activation function, we use the ${\text{Re}} LU( \cdot )$ function here. $W^{(l)}$ is the weight matrix of layer l. $b^{(l)}$ is the intercept of layer l. Normalize Formula (3), and Formula (4) can be obtained.

4 $$X^{(l + 1)} = \sigma \left( {D^{ - 1} AX^{(l)} W^{(l)} + b^{(l)} } \right),$$

where D is the degree matrix, and the process of normalization is realized by the degree matrix. $D^{ - 1} A$ is the normalization. Symmetric normalization is carried out in the basis of normalization, and Formula (5) is obtained.

5 $$X^{(l + 1)} = \sigma \left( {D^{{ - \frac{1}{2}}} AD^{{ - \frac{1}{2}}} X^{(l)} W^{(l)} + b^{(l)} } \right)$$

Specifically, if we ignore the intercept, and we use the corresponding adjacency matrix G to construct a network, and we set the layerwise propagation rule of GCN as:

6 $$X^{(l + 1)} = \sigma \left( {D^{{ - \frac{1}{2}}} AD^{{ - \frac{1}{2}}} X^{(l)} W^{(l)} } \right)$$

By deploying GCN on the constructed heterogeneous map to combine node similarity and directly linked association information, we construct an encoder based on GCN to learn the low-dimensional representations of diseases and miRNAs.

Therefore, we define $\mu$ as penalty factor, and we use it to control the contribution of similarity to GCN propagation [17]. The input graph we set is:

7 $${\text{A}} = \left[ {\begin{array}{*{20}c} {\mu \sim S^{m} } & N \\ {N^{T} } & {\mu \sim S^{d} } \\ \end{array} } \right],$$

where $\mu$ is a penalty factor. $S^{m}$ is the similarity matrix of miRNA and $S^{d}$ is the similarity matrix of disease. N is the miRNA-disease associations matrix.

And we initialize X as:

8 $$X^{(0)} = \left[ {\begin{array}{*{20}c} 0 & N \\ {N^{T} } & 0 \\ \end{array} } \right]$$

Then, we define the first layer formula of the GCN encoder as:

9 $$X^{(1)} = \sigma \left( {D^{{ - \frac{1}{2}}} AD^{{ - \frac{1}{2}}} X^{(0)} W^{(0)} } \right),$$

where $X^{(1)}$ is the first layer embedding of miRNA and disease nodes in heterogeneous networks. And $W^{(0)}$ is the $W^{(l)}$, which we put forward in Formula (3). In Formula (9), l = 1, it represents the first layer embedding of heterogeneous networks. In the process of embedding, after many experiments, we finally decided to adopt three-layer convolution layers, and the input of each layer is the output of the previous layer.

The pseudocode is shown in Table 1.

**Table 1:** The pseudocode

**Input:**
miRNA–miRNA similarities matrix, disease–disease similarities matrix, adjacent matrix A;
Construct the input graph G = (V,E)
**Output:**
Initialize embedded dimension, learning rate, training epoch, dropout rates;
Initialize embeddings;
Iterate according to the layerwise propagation rule of GCN;
Introduce an attention mechanism;
Combine other embeddings and obtain final embeddings of miRNAs and diseases;
Obtain the Loss function

### Optimization

In our datasets, we set the number of miRNAs to A and the number of diseases to B. Then we take the miRNA- disease association data as positive examples and the others as negative examples. However, due to the influence of miRNA and the number of disease associations, no association was observed in miRNA-diseases, so we set the weighted cross-entropy as the loss function. The loss function is:

10 $${\text{Loss}} = - \frac{1}{A \times B}\left( {\lambda \times \sum\nolimits_{{(i,j) \in y^{ + } }} {\log a_{ij}^{{\prime }} } + \sum\nolimits_{{(i,j) \in y^{ - } }} {\log \left( {1 - a_{ij}^{{\prime }} } \right)} } \right),$$

where $(i,j)$ represents the pair of miRNAs $m_{i}$ and disease $d_{j}$. And $y^{ + }$ represents the positive instances sets and $y^{ - }$ represents the negative instances sets. $\lambda { = }\frac{{|y^{ - } |}}{{|y^{ + } |}}$, and it can limit the influence of data imbalance.

### Layer attention

Attention mechanism is a broad concept, which means that people or machines selectively pay attention to information with different degrees of importance and process the information. Attention mechanism has different functions, types and scope of application.

Starting from the working mechanism of attention mechanism, attention methods can be summarized into two categories: selective attention mechanism and self-attention mechanism.

Selective attention mechanism is an explicit attention method, which obtains the attention weight by predicting the importance of each part of the data to the task optimization goal. Then the attention weight is used to explicitly enhance the important components of the data and suppress the components of the data that have nothing to do with the task optimization objectives.

Selective attention mechanism is widely used in the processing and analysis of language signals, visual signals and other information, which greatly improves the nonlinear expression ability of neural network and the abstraction ability of high-level semantics. The selective attention mechanism takes the data itself as input, uses neural network learning to generate an attention mask as a result of predicting the importance of each part of the data, next uses the attention mask to enhance or suppress the feature.

Self-attention mechanism is an attention method that aligns internal information observation with external information observation so as to improve the accuracy of local feature expression. The core of the self-attention mechanism is the idea of "non-local average, that is, we first find the feature expression that is close to the target position in the non-local region, next make use of the similarity between the target location information expression and other non-local region information expression to achieve information transmission in the non-local region by weighted addition, and modify the information expression of the target location.

The introduction of attention mechanism improves the data processing and analysis ability of the model. It is of great significance to improve the performance of the model.

**Fig. 1:**
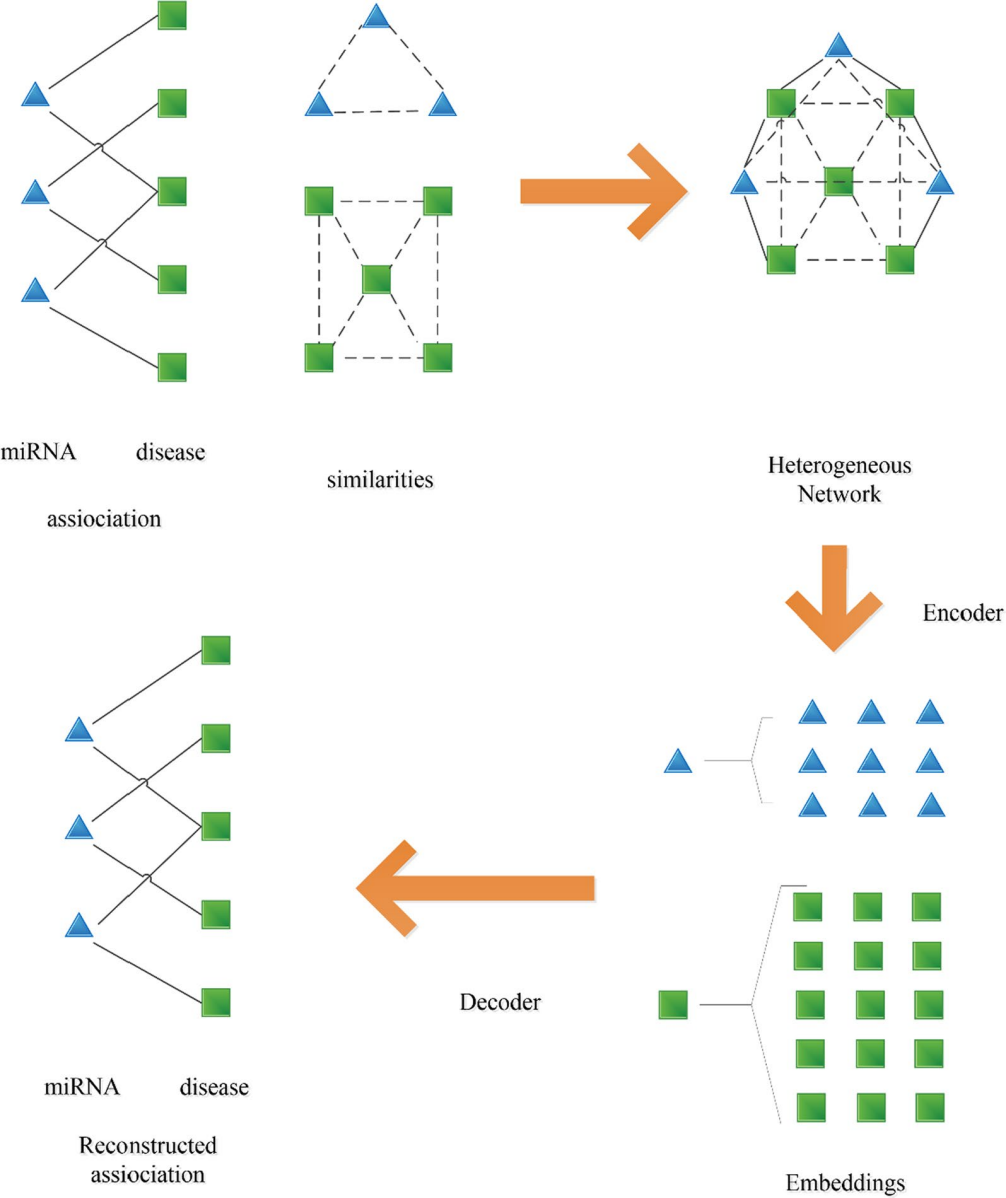
The workflow of LAGCN

Next, we will introduce layer attention graph convolutional network (LAGCN), such as in Fig. 1.

As shown in Fig. 1, triangles represent miRNA, squares represent disease. We first use the known associations between miRNA and disease, the known association between miRNA-miRNA and the association between disease-disease to build heterogeneous networks, next through a series of operations such as coding and decoding to get new miRNA-disease associations.

MiRNA and disease build three networks. Next these three networks form a heterogeneous network. After through process of encoding and decoding, we can get the association between miRNA and disease.

### Datasets

Our integrated datasets contain 5430 miRNA-disease associations between 495 miRNA and 383 diseases. Detailed datasets on miRNA and disease can also be downloaded from HMDD. HMDD is a database that collects evidence of a link between human microRNA (miRNA) and disease supported by experiments.

At present, there are 572 miRNA genes, 378 diseases in HMDD v2.0. In this paper, our datasets are from HMDD v2.0. Our associated data sets are from [18, 19].

We define $MSI$ as the miRNA functional similarity. For details on the definition and description of the related formulas of $MSI$, please read the reference [20].

Next we make use of a directed acyclic graph (DAG) to represent diseases, and DAG structure can be used to calculate the similarities between diseases.

For a disease d, we set $DAG\left( d \right) = \left( {E\left( d \right),e\left( d \right)} \right)$ to represent a hierarchical relationship. For details on the definition and description of the related formulas of the semantic of disease and semantic similarity, please read the reference [20].

For details on the definition and description of the related formulas of similarities between miRNAs and diseases, please read the reference [18, 21].

## Results

In our experiment, a fivefold-cross-validation(5-cv) method is used to predict the performance of this method. First of all, we randomly divide the known miRNA-disease association into five subsets of equal size, each subset takes turns as the test set, and the remaining four subsets as the training set, and the process of cross-validation is repeated five times. Although we introduce many evaluation criteria, for the accuracy of the results, we use AUC as the main evaluation criteria, because the metric can evaluate the superiority of the model without any specific threshold. Of course, several other metrics are also within our consideration.

Next, we will introduce some hyperparameters in the layer attention graph convolutional network, $emb\_dim$ represents embedded dimension, $lr$ represents the initial learning rate of the optimizer, $epoch$ represents the training epochs of the layer attention graph convolutional network, $adjdp$ represents the node dropout which is one of the two dropout rates, and $dp$ represents the other, and the other is a regular dropout. We also introduce the penalty factor $simw$ in heterogeneous networks. In order to get better results, we have carried out many experiments and repeatedly adjusted the values of various parameters. Finally, select the value of each hyperparameter as follows: $dp \in \left\{ {0.1,0.2,0.3,0.4,0.5} \right\}$, $adjdp \in \left\{ {0.1,0.2,0.3,0.4,0.5,0.6} \right\}$ $simw \in \{ 2,4,6,8\}$, At last, we set $epoch = 250$, $emb\_dim = 64$, $lr = 0.01$, $adjdp = 0.6$, $dp = 0.4$, $simw = 6$. At this time, AUC = 0.9091, recall = 0.5421, accuracy = 0.9689. The reason for $epoch = 250$ is that the values of AUC are relatively better at this time, and the results of other indicators are also not bad.

LAGCN make uses the heterogeneous network of miRNA-disease to construct a predictive model. we can train the layer attention graph convolutional network on different heterogeneous networks according to different miRNA-miRNA similarities. Next, we discuss the effect of these miRNA-miRNA similarities on the performance of LAGCN.

Through 5-cv of heterogeneous networks with miRNA-miRNA similarities, the evaluation of LAGCN model is completed, and it is concluded that LAGCN is reliable regardless of similarity measurement and miRNA characteristics.

We try to construct a network only by miRNA-disease associations, so we construct a stripped-down LAGCN based on the network. After many experiments, we find that the simplified LAGCN has lower AUC scores than the original model, which indicates that miRNA-miRNA similarities and disease-disease similarities in heterogeneous networks are useful for this method and lead to the improvement of LAGCN performance.

### Evaluation metrics

In this paper, we decide to use AUC, recall and accuracy as metrics to evaluate the prediction performance of the model.

The number of positive samples predicted correctly is expressed by TP, the number of correctly predicted negative samples is expressed by TN, the number of predicted incorrectly positive samples is expressed by FP, and the number of predicted incorrectly negative samples is expressed by FN.

The calculation formula for various evaluation metrics are as follows:

11 $$recall = \frac{TP}{{TP + FN}}$$

12 $$accuracy = \frac{TP + TN}{{TP + TN + FP + FN}}$$

AUC represents the area under the receiver–operating characteristic curve [18].

**Fig. 2:**
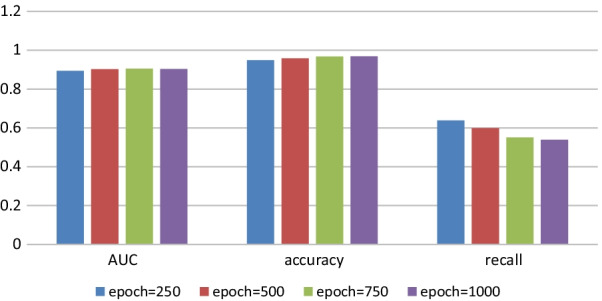
The value of evaluation metrics when epoch takes different values

We have adjusted the values of each parameter several times, and when the $epoch$ takes different values, $emb\_dim = 64$, $lr = 0.01$, $adjdp = 0.6$, $dp = 0.4$, $simw = 6$. The values of each evaluation metric are shown in Fig. 2.

As we can be from Fig. 2, with the increase of the epoch, the values of AUC and accuracy increase slightly though the impact is not significant, while the value of recall decreases with the increase of epoch. Because the higher the value of epoch in the program, the greater the running time and memory consumption, so we chose the value of epoch value of 250 for subsequent experiments.

**Fig. 3:**
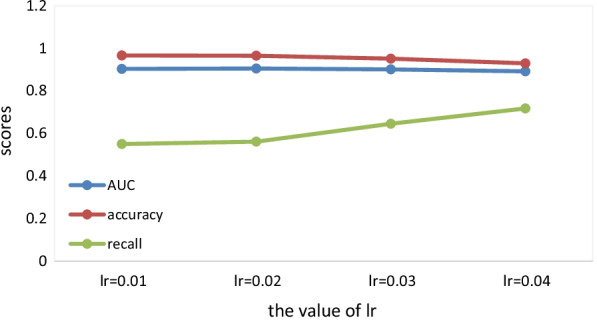
The comparison of evaluation metrics when lr takes different values

When the $lr$ takes different values, $epoch = 250$, $emb\_dim = 64$, $adjdp = 0.6$, $dp = 0.4$, $simw = 6$, the values of each evaluation metric are shown in Fig. 3.

We can find that with the increase of $lr$,the value of recall increases, but the values of AUC and accuracy decrease greatly. Therefore, in order to optimize the final results, we finally decide to take a smaller value of $lr$.

**Fig. 4:**
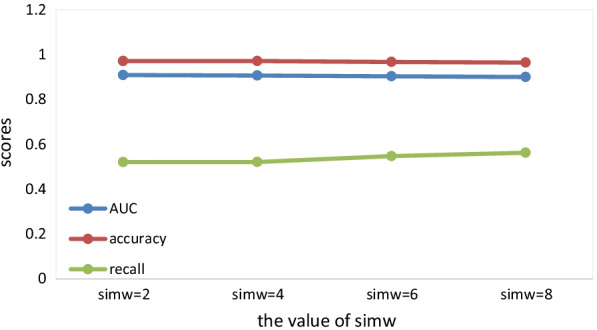
The value of evaluation metrics when simw takes different values

When the $simw$ takes different values, $epoch = 250$, $emb\_dim = 64$, $lr = 0.01$, $adjdp = 0.6$, $dp = 0.4$, the values of each evaluation metric are shown in Fig. 4.

We can find that with the increase of $simw$, the values of AUC and accuracy change little, but the value of recall increases. Therefore, in order to optimize the final results, we finally decide to take a higher value of $simw$.

**Fig. 5:**
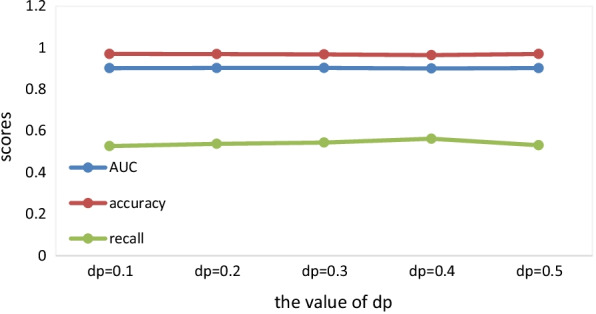
The value of evaluation metrics when dp takes different values

When the $dp$ takes different values, $epoch = 250$, $emb\_dim = 64$, $lr = 0.01$, $adjdp = 0.6$, $simw = 6$, the values of each evaluation metric are shown in Fig. 5.

Among the multiple metrics, the value of AUC is the most important, so we take AUC as the main metric.

We conducted many experiments and finally selected the relatively optimal result.

**Fig. 6:**
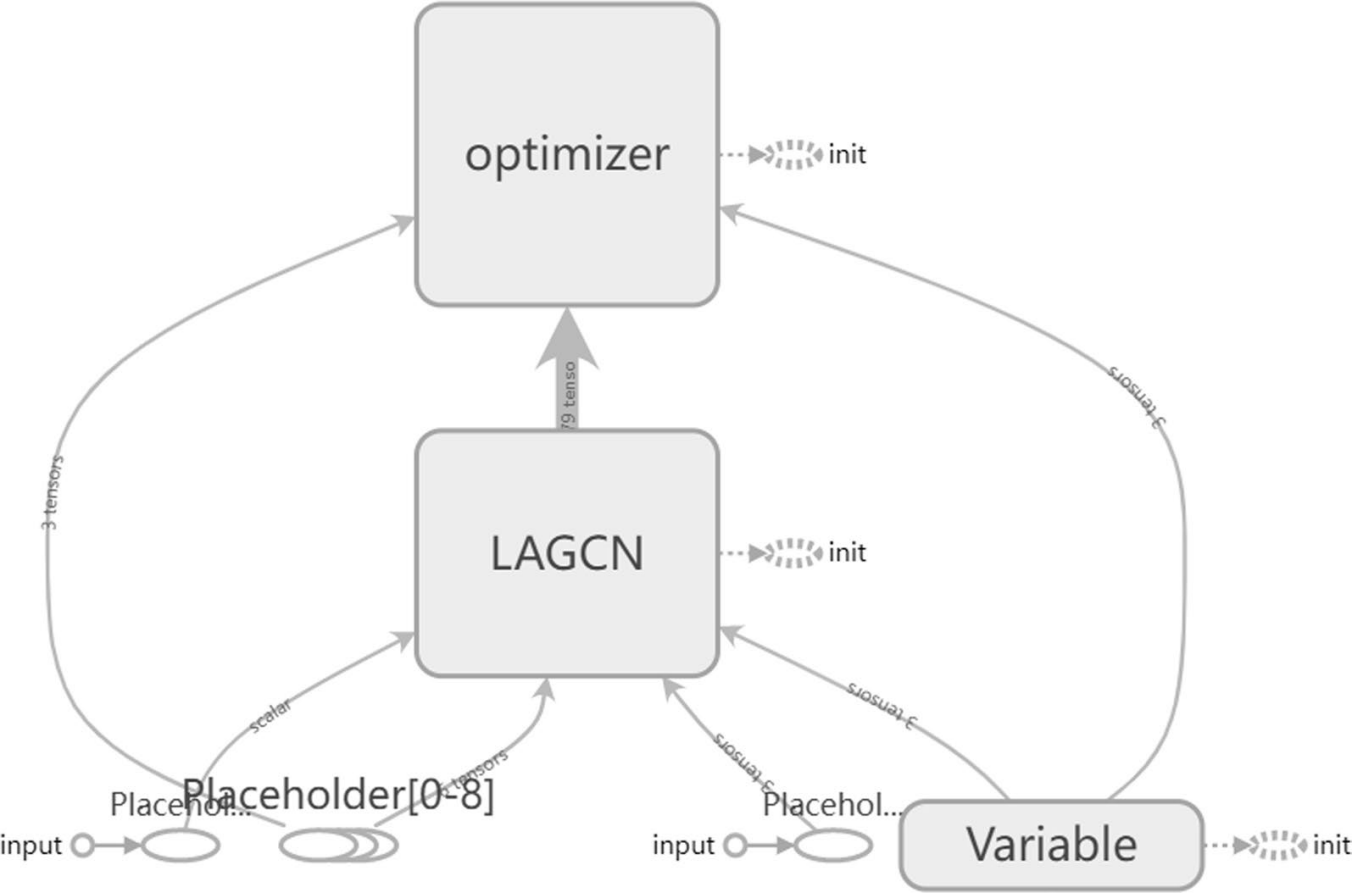
The network structure

Then, we use the Tensorboard tool to visualize the network structure of our model (Fig. 6.).

## Discussion

In order to evaluate the performance of LAGCN, we chose six state-of-the-art methods for comparison. These methods are the best methods in the direction of disease association prediction at present. Next, we compare the proposed method with other methods. The comparison methods used in the experiment include HGIMDA, RLSMDA, HDMP, WBSMDA, RWRMDA and ICFMDA [22]. The values of various methods are shown in Table 2.

**Table 2:** The AUC of various methods

Method	AUC
LAGCN	0.9091
HGIMDA	0.8077
RLSMDA	0.6953
HDMP	0.7702
WBSMDA	0.8031
RWRMDA	0.7891
ICFMDA	0.8519

In addition, it can still be used in other datasets. For example, Zhou et al. used similar methods to achieve good results in drug correlation experiments.

## Conclusions

In this paper, we introduced the method of LAGCN to identify potential miRNA-disease associations. Different from the existing methods of using the bipartite graph, LAGCN captures the topological information of heterogeneous networks composed of miRNA-disease association, miRNA-miRNA similarities and disease-disease similarities. By adaptively combining embedding and attention mechanisms at different convolution levels, LAGCN has achieved good performance in predicting miRNA-disease associations, and it is superior to other association prediction methods and baseline methods.

We think that find an appropriate way to predict the potential associations between miRNAs and diseases is important, which can improve our understanding of the disease and humans themselves, and promote the cure of the disease in some ways.

LAGCN is a good method for predicting miRNA-disease similarities, but it also has some problems, such as over-smoothing. we will continue studying and finding some ways to solve these problems.

## Data Availability

The miRNA-diseasse database analyzed in the study is from HMDD v2.0, http://www.cuilab.cn/hmdd.
